# Early Growth Response Factor 4 (EGR4) Expression in Gut Tissues and Regional Lymph Nodes of Cattle with Different Types of Paratuberculosis-Associated Lesions: Potential Role of EGR4 in Resilience to Paratuberculosis

**DOI:** 10.3390/ani15071012

**Published:** 2025-03-31

**Authors:** Alejandra Isabel Navarro León, Marta Alonso-Hearn, Marta Muñoz, Natalia Iglesias, Gerard Badia-Bringué, Tania Iglesias, Ana Balseiro, Rosa Casais

**Affiliations:** 1 Center for Animal Biotechnology, Servicio Regional de Investigación y Desarrollo Agroalimentario (SERIDA), 33394 Deva, Spain; alejandraisabel.navarroleon@asturias.org (A.I.N.L.); marta.munozllamosas@asturias.org (M.M.);; 2 Animal Health Department, NEIKER, Basque Institute for Agricultural Research and Development, Basque Research and Technology Alliance (BRTA), 48160 Alava, Spain; malonso@neiker.eus (M.A.-H.); gbadia@neiker.eus (G.B.-B.); 3 Unidad de Consultoría Estadística, Servicios Científico-Técnicos, Universidad de Oviedo, Campus de Gijón, 33203 Gijón, Spain; 4 Departamento de Sanidad Animal, Facultad de Veterinaria, Universidad de León, 24071 León, Spain; 5 Instituto de Ganadería de Montaña (IGM, CSIC-ULE), 24346 León, Spain

**Keywords:** EGR4, bovine paratuberculosis, immunohistochemistry, PTB resilience, PTB control

## Abstract

Summary-data-based Mendelian randomization (SMR) analysis identified a novel cis-expression quantitative loci (cis-eQTL) associated with the upregulation of the expression of the early growth response factor 4 (EGR4) gene in animals with paratuberculosis (PTB)-associated multifocal lesions, which has been suggested to be modulating the NF-kβ-induced proinflammatory immune response to *Mycobacterium avium* subsp. *paratuberculosis* (Map) infection. To confirm these findings and to study the role of EGR4 expression in PTB resilience, the number of EGR4-expressing cells were analysed in paraffin-fixed gut tissues and regional lymph nodes of naturally Map-infected Holstein Friesian cows with focal, multifocal (subclinical and clinical), and diffuse lesions (intermediate and multibacillary), and in controls without lesions by quantitative anti-EGR4 immunohistochemistry. Subclinical animals with multifocal lesions showed a significantly higher number of EGR4-positive cells and were sacrificed at a significantly older average age than the remaining groups (*p* < 0.001 in all cases). We hypothesize that EGR4 could be mitigating the negative impact of Map infection on host clinical status through its involvement in three molecular mechanisms that promote resilience: (i) limiting NF-kβ-mediated proinflammatory responses, (ii) controlling tissue damage, acting as a brake on T-cell proliferation and cytokine production, and (iii) favouring tissue repair through interaction with epidermal growth factor receptor (EGFR).

## Simple Summary

Bovine paratuberculosis (PTB), caused by *Mycobacterium avium* subsp. *Paratuberculosis* (Map), has a high impact on animal welfare and on the dairy industry. The selective breeding of PTB-resilient animals might be an effective strategy for producers to maintain a healthy and productive herd in a sustainable and cost-effective manner. A disease-resilient animal can be defined as a Map-infected animal that remains asymptomatic over its productive life without major variations in their health and milk production. This study investigated the role of early growth response factor 4 (EGR4), a protein that modulates immune responses and promotes tissue repair, in cattle resilient to PTB. Specifically, the number of cells that expressed EGR4 in the gut tissues of cattle with PTB-associated histological lesions with increased severity (focal, multifocal, and diffuse) were quantified. Animals with multifocal lesions and no clinical signs had significantly higher numbers of EGR4-positive cells and live longer compared to those with diffuse lesions and clinical signs. This increase in the number of EGR4-positive cells was associated with a reduction in the fitness cost caused by PTB and a slower disease progression.

## 1. Introduction

Bovine paratuberculosis (PTB), caused by *Mycobacterium avium* subsp. *paratuberculosis* (Map), is a chronic granulomatous enteritis responsible for important economic losses in the dairy industry worldwide [1,2]. Map has a zoonotic potential, as it has been postulated as a possible trigger factor in several human autoimmune diseases such as Crohn’s disease [3,4], rheumatoid arthritis [5,6], multiple sclerosis [7], and type I diabetes [8].

PTB is an important infectious veterinary disease in food-producing animals, which is difficult to control. Current PTB control programs are based on the identification and culling of positive animals and the implementation of good management practices [9,10,11]. However, such programs are strongly conditioned by the low sensitivity of the current diagnostic techniques for the detection of early subclinical infections. In this context, the identification and selective breeding of PTB-resilient animals might be crucial, as a complementary strategy, to improve PTB control [12,13]. The selection of PTB-resilient animals might help to improve herd productivity and animal welfare, allowing producers to maintain a healthy and productive herd in a sustainable and cost-effective system. Resilient animals use three defence strategies: avoidance, tolerance, and resistance [14]. Avoidance refers to behaviours which reduce interactions with diseased animals, limiting the risk of exposure to infectious agents; although, in the context of PTB, animals do not exhibit any known behaviour to avoid Map infection. Resistance refers to the elimination of the pathogen preventing pathogen invasion, induction of the innate immune response and inflammation, limiting the number of bacteria when the infection is established. Finally, disease tolerance refers to the limitation of the tissue damage or protection of cells from damage caused by the infectious agent, enhancing tissue repair and mitigating the negative impact of infection on host fitness without directly affecting the pathogen burden [15,16]. Thus, the immune system protects the host against infections primarily by detecting and eliminating the invading pathogen, but it can also reduce the negative impact of infection on host fitness.

PTB-associated lesions in cattle have been classified according to their severity and extension as focal, multifocal, and diffuse [17]. Genome-wide association studies (GWASs) have allowed the identification of single-nucleotide polymorphisms (SNPs) associated with these three pathological outcomes in Holstein Friesian cattle using whole-genome sequence (WGS) data analysis [18]. Most of the identified SNPs were located in non-coding regions of the genome that contained intergenic and intronic regions enriched in regulatory elements, indicating that those genetic variants exert their effects through the modulation of gene expression. In fact, 192 and 92 SNPs defining thirteen and nine different functional mutations or quantitative trait loci (QTLs) were highly associated (*p* ≤ 5 × 10^−7^) with the multifocal (heritability = 0.075) and the diffuse (heritability = 0.189) lesions, respectively. No common SNPs were found for the two pathological outcomes. Some of these QTLs overlapped with QTLs previously associated with PTB susceptibility.

Summary-data-based Mendelian randomization (SMR) identified gene expression regulatory polymorphisms associated with PTB by the modulation of the nuclear factor kappa β (NF-kβ)-mediated inflammatory response [19]. Specifically, a novel cis-eQTL that regulates the expression of the early growth response factor *4* (*EGR4*) gene was identified. This specific cis-eQTL showed pleiotropic association with animals with PTB-associated multifocal lesions (*p* = 0.002), i.e., the upregulation of EGR4 expression levels correlated with the presence of multifocal lesions. EGR4 might be limiting the NF-kβ-mediated inflammatory response to Map infection, thus preventing an exacerbated immune response and tissue damage. Hence, EGR4 could be mitigating the negative impact of Map infection on host fitness through its involvement in resilience molecular mechanisms.

The early growth response (EGR) family of zinc-finger transcription factors includes four members (EGR1-4), which are activated by extracellular stimuli and various mitogenic signals such as growth and differentiation signals, tissue injury, and apoptotic signals on different cell types, including lymphocytes and epithelial cells [20,21,22]. EGR proteins are expressed in distinct cell types and regulate the transcription of a wide range of genes, including those involved in cell growth control and apoptosis [23]. In T cells, the immediate-early EGR zinc-finger proteins act as transcriptional regulators of inflammatory genes, particularly during the initial phase of T-cell activation. Moreover, they have also been involved in central and peripheral immunotolerance [24,25]. Both processes play a crucial role in regulating the immune response, even during an infection, which is particularly significant in persistent bacterial infections, such as those caused by Map, where prolonged inflammation can result in significant damage to host tissues if it is not properly regulated.

The EGR4 zinc-finger protein was first described in the central nervous system [26]. Since then, it has been demonstrated that EGR4 expression can occur in other locations. For example, EGR4 has also been isolated in human and mouse testis, localized in stem cells, and the most undifferentiated spermatogonial cells, where it is involved in specific biological processes such as spermatogenesis [27,28]. Moreover, EGR4 has been detected in low levels in mammary epithelial cells of different species [29], as well as in colorectal cancer-associated fibroblasts [30], among other tissues, even being related to the proliferation of cancer cells [31]. On the other hand, EGR4 has shown promising activity in regulating immune responses and promoting tissue repair due to its stable physical interaction with immune–inflammatory mediators NF-κβ p50 and p65, which shows the ability of EGR4 to interact with NF-kβ and control the transcription of genes encoding inflammatory cytokines [32]. This suggests that EGR4 modulates the NF-κB-induced proinflammatory response, being a key regulator of T-cell differentiation and function. Specifically, these authors showed that EGR4 is rapidly upregulated upon T-cell receptor engagement, serving as a critical “brake” on T-cell activation [33]. They also showed that the suppression of Ca^2+^ signals by EGR4 controls Th1 differentiation and anti-cancer immunity in vivo [33]. Taking that into consideration, we hypothesized that the upregulation of EGR4 could serve as a brake of T-cell activation and inflammatory cytokine production through interaction with NF-kβ, preventing the excessive exacerbation of immune responses and tissue damage, and therefore promoting disease resilience in subclinical Map-infected animals with multifocal lesions. In addition, EGR4 interacts with epidermal growth factor receptor (EGFR) [34], which could favour tissue repairing. Hence, EGR4 could be mitigating the negative impact of Map infection on host clinical status through its involvement in molecular mechanisms of resilience.

The aim of this study was to evaluate the expression of the EGR4 in gut tissues of animals with PTB-associated histological lesions with increased severity and controls without lesions to confirm the SMR results, where the upregulation of EGR4 expression levels was correlated with the presence of multifocal lesions. For this purpose, the number of EGR4-expressing cells per µm^2^ was analysed in a paraffin-fixed ileocecal valve (ICV), ileocecal lymph nodes (ICVLNs), distal jejunum (DJE), and jejunal lymph nodes (JELNs) of animals with focal (n = seven), multifocal (n = twelve, seven animals without and five with PTB-associated clinical signs), and diffuse lesions (n = fourteen), and controls without lesions (n = six), by immunohistochemistry (IHC) using a rabbit polyclonal anti-EGR4 antibody. Here, these results are presented and the relation of EGR4 levels with resilience to PTB is discussed.

## 2. Materials and Methods

### 2.1. Animals and Samples

Thirty-nine Holstein Friesian cows (n = thirty-nine) from farms located in the Principality of Asturias (northwest of Spain) were used for this study. Samples of blood, faeces, and tissues from all the animals were collected and processed, as previously described [35]. Tissue samples (ICV, ICVLN, DJE, and JELN) were collected from the slaughtered animals in situ at the local abattoir after evisceration. The Map infection status of the animals was determined by histopathological analysis, specific anti-Map antibody serum ELISA testing (IDEXX, Montpellier, France), and bacteriological culture and specific real-time PCR using the LSI VetMAX^TM^ Triplex *M. paratuberculosis* kit (Life technologies, Lissieu, France) on tissues and faeces, following the procedures described by Blanco-Vazquez et al. (2020) [35]. The presence or absence of PTB-associated clinical signs was also registered.

### 2.2. Tissue Preparation and Histopathological Classification of Animals

Samples were fixed in 10% neutral buffered formalin, sliced, and embedded in paraffin blocks. Tissue sections (4 µm) were cut and placed on microscope slides (Superfrost Plus, Menzel GmbH, Braunschweig, Germany), and dried at 37 °C for 24 h. Hematoxylin-eosin and Ziehl–Neelsen staining were used to carry out the PTB histopathological diagnosis and confirm the presence of acid-fast bacteria, respectively. Slices were analysed using an Olympus BH-2 light microscope (Olympus, Tokyo, Japan). Histological lesions associated with bovine PTB were classified according to González et al. (2005) [17]. Four complementary target sections of the gut tissue (DJE, ICV, ILN, and JELN) were examined and the animals were classified as focal, multifocal, diffuse, or without lesions, based on the presence/absence and the type of PTB-associated histological lesions. Diffuse lesions were further subdivided into diffuse lymphoplasmacytic or paucibacillary, diffuse intermediate, and diffuse histiocytic or multibacillary [36].

### 2.3. Single Anti-EGR4 Immunohistochemistry (Anti-EGR4 IHC)

To investigate EGR4 expression, the number of cells expressing EGR4/µm^2^ within the DJE, JELN, ICV, and ILN of animals with different types of PTB-associated lesions and in negative control animals without lesions were quantified by single anti-EGR4 IHC. The effect of the presence of clinical signs in animals with multifocal lesions and the subtype of the diffuse lesion (intermediate and multibacillary) on EGR4 expression was also evaluated. The study was performed considering four (control, focal, multifocal, and diffuse) or six (control, focal, multifocal without clinical signs, multifocal with clinical signs, diffuse intermediate, and diffuse multibacillary) groups or categories.

Formalin-fixed paraffin-embedded DJE, JELN, ICV, and ICVLN samples were cut into 3 µm sections and placed on microscope slides (Superfrost Plus, Menzel GmbH, Braunschweig, Germany). Sections were dewaxed and rehydrated using tap water at room temperature (RT), followed by antigen retrieval for epitope unmasking by incubation with sodium citrate tribasic dihydrate 0.1% (Sigma-Aldrich, St. Louis, MO, USA) dissolved in preheated tris-buffered saline (PBS) containing 1% Triton X-100 (Sigma-Aldrich, St. Louis, MO, USA) for 20 min at 95 °C. Endogenous peroxidase activity was blocked by treating the slides with 3% hydrogen peroxide (Sigma-Aldrich, St. Louis, MO, USA) in methanol (VWR, Monroeville, PA, USA) for 10 min at RT. Slides were washed with tap water at RT, and then non-specific binding was blocked using 10% normal goat serum (Vector Laboratories) containing 3% bovine serum albumin (BSA, Sigma-Aldrich, St. Louis, MO, USA) for 15 min at RT. For EGR4 detection, a rabbit polyclonal antibody raised against a synthetic peptide from the carboxy terminus of human EGR4 (LS-B1525, LifeSpan Bioscience, Seattle, WA, USA) was used. The BLAST analysis showed 100% identity with the bovine protein. Tissue sections were incubated with a 1:100 dilution of anti-EGR4 antibody overnight at 4 °C and then washed three times with TBS 1X (TBS 1X, 5 mM Tris (Merck KGaA, Darmstadt, Germany)/HCl (Panreac Química, SLU, Barcelona, Spain) pH 7.6, 136 mM NaCl (Merck KGaA, Darmstadt, Germany) at RT. After that, sections were incubated for 30 min at RT with a biotinylated anti-rabbit IgG secondary antibody produced in goat serum (Vector Laboratories, Burlingame, CA, USA) at 1:200 dilution, and slides were washed as previously described. For signal detection sections were incubated for 30 min at RT with ABC kit Peroxidase (Avidin-Biotin Complex kit PO) Standard (Vector Laboratories) following the manufacturer’s instructions, and then they were washed three times with TBS 1X and incubated with the peroxidase substrate 3,3′-Diaminobenzidine tetrahydrochloride (DAB) (Sigmafast, Sigma-Aldrich, St. Louis, MO, USA) for 3 min at RT, controlling colour development by observation under the microscope. Afterwards, samples were rinsed with tap water for 5 min and counterstained in Mayer’s hematoxylin (MerckKGaA, Darmstadt, Germany) for 20 s before washing, dehydrating, and mounting with DPX (Merck KGaA, Darmstadt, Germany). In the IHC analysis of the bovine experimental samples, a negative control, performed with the omission of the primary antibody, was included in each IHC run to confirm the specificity of the primary antibody. Since pigments such as hemosiderin in lymph nodes can be interpreted erroneously as positive immunolabelling when DAB is used as a chromogen, the above-mentioned negative controls were essential to differentiate granules from EGR4-positive staining.

Before carrying out anti-EGR4 IHC in the experimental samples, a preliminary IHC assay was performed to assess the performance of the anti-EGR4 antibody, checking EGR4-positive staining and its associated staining pattern using mouse testis as a positive control tissue for EGR4-positive expression [27,37]. Negative and positive controls performed with or without omission of the primary antibody, respectively, were included in the preliminary assay performed in mouse testis (Figure S1). Negative controls omitting the primary antibody were also performed on all four bovine tissue sections studied.

### 2.4. Image Acquisition, Quantification, and Cell Counting Procedure for EGR4 Immunohistochemistry

For image acquisition, the DAB-labelled sections were examined using an Olympus BH-2 light microscope (Olympus, Tokyo, Japan) and acquired with an Olympus DP-12 digital camera (Olympus, Tokyo, Japan). Images for the quantification of EGR4-expressing positive cells were always taken within the mucosa area for ICV and DJE (including mucosa-associated lymphoid tissue (MALT), where PTB-associated lesions are also commonly found), whereas the entire histological section was used for JELNs and ICVLNs. Areas containing preparation artefacts, cell debris, or the edges of the slide were avoided during image acquisition.

The expression of EGR4 was evaluated in cattle with different histopathological forms of PTB and control animals without lesions by single EGR4-IHC, counting the number of EGR4-positive labelled cells per µm^2^ in ten randomly selected fields/images (representative of the different tissue areas; for instance, for ICV, this includes the apical and basal laminal propria and lymphoid tissue areas) per individual and tissue section (ICV, ICVLN, DJE, and JELN) at a final magnification of 400× (i.e., 40 images/measurements of the number of positive cells per µm^2^ for each individual in a group). Each image (1600 × 1200 pixels) had a total surface area of 41,207.52 µm^2^ (234.4 µm length × 175.8 µm height). The number of EGR4-positive labelled cells per µm^2^ were automatically counted in each selected image using open-source QuPath software version 0.4.3 [38]. Briefly, the number of EGR4 positive cells per µm^2^ were quantified by applying a script developed at the Image Processing Unit of the University of Oviedo, to the randomly selected fields. During the initial training phase, which was used to show the program the cells that have to be considered positive and negative, images were analysed using the QuPath ‘Estimate stain vectors’ command to enhance stain separation. Manual positive and negative cell counts were performed with the ‘Positive cell detection’ command in representative areas to fine-tune threshold parameters, ensuring the software detected cells correctly. Afterwards, for the specific detection of EGR4-labeled positive cells, a script incorporating the stain vectors and threshold values from the initial training was utilized for image analysis. A test phase was performed before the final image analysis. The counting results were revised by an expert histopathologist.

### 2.5. Statistical Analysis

Statistical analysis was performed using R software (R Development Core Team, version 4.1.3). To compare differences in the quantitative variables between two groups, a Student’s t-test or Welch’s test was used. For comparisons with three or more groups, the Kruskal–Wallis test was employed. When the Kruskal–Wallis test yielded statistically significant results, the post hoc Dunn’s test was conducted to determine which specific pairwise groups had statistically significant differences. The level of significance was set at *p* < 0.05. Results were expressed as the mean ± standard deviation (SD) or the median (25th percentile to 75th percentile) of the number of positive cells per µm^2^ for parametric and non-parametric post hoc test analysis, respectively.

Moreover, a multivariate linear model considering both variables, i.e., age and histopathological group, as predictors of the total number of positive cells per µm^2^ in the four tissue sections, with the effect of the group adjusted by the age, was generated. The analysis was performed considering both four and six categories.

## 3. Results

### 3.1. Map Infection Status

Animals (n = 39) were classified according to the type of histological lesions present in their gut tissues and regional lymph nodes [17], into three groups with increasing severity: focal (n = seven), multifocal (n = twelve), and diffuse (n = fourteen), which was further subdivided into diffuse intermediate (n = seven) and multibacillary (n = seven). The multifocal group was divided into two subgroups to investigate whether EGR4 expression levels could be related to the presence (n = five, 41.67%) or absence (n = seven, 58.33%) of clinical signs (diarrhoea, weight loss, decreased milk production), assuming that their presence suggests no control of disease progression. In addition, a healthy control group (n = six) of animals without PTB-associated histological lesions, and with a negative ELISA test, bacteriological culture, and real-time PCR of tissues and faeces, was included. Information on the infection status, age, and presence of clinical signs of each animal is shown in Table 1.

Map-infected animals with focal lesions which did not show clinical signs had a 100% positive real-time PCR of gut tissues, 42.86% positive Ziehl–Neelsen (ZN) test, and tissue bacteriological culture with no presence of anti-Map antibodies detected. Animals with multifocal lesions were 100% positive by ZN testing, 8.33% positive by anti-Map antibodies ELISA, and 30.00% and 66.66% positive by faeces and tissues real-time PCR, respectively. If we analysed animals with multifocal lesions without clinical and with clinical signs separately (without clinical signs/with), 100%/100% were positive by ZN testing, 0%/20% positive by anti-Map antibodies ELISA, 16.66%/50% positive by faeces qPCR, 71.42%/60% by tissues qPCR, and 0%/0% and 28.57%/20% positive by faeces and tissue bacteriological culture. Animals with diffuse intermediate and multibacillary lesions were 100% positive by ZN testing, anti-Map antibodies ELISA, and tissues real-time PCR, 85.71% and 100% positive by faeces PCR, 28.57% and 42.87% positive by faeces bacteriological culture, and 85.71% and 100% positive by tissues culture, respectively. As expected, animals with diffuse multibacillary PTB-associated histological lesions had higher percentages of positivity. All the animals with diffuse lesions but one with diffuse intermediate lesions (92.30%) showed clinical signs.

### 3.2. Assessment of the Specificity of the Immunoreagents Used in the Single-EGR4 Immunohistochemistry Assay

The enrichment of EGR4 expression in cattle with PTB-associated multifocal lesions was investigated in intestinal tissues and regional lymph node samples (DJE, JELN, ICV, and ICVLN) by anti-EGR4 IHC and the quantification of the number of EGR4-positive cells per µm^2^ in animals with different types of PTB-associated lesions (n = 33) and control animals without lesions (n = 6). The IHC procedure and the reagents used (primary and secondary antibodies, DAB labelling, etc.) were initially evaluated using mice testis as the positive control tissue for EGR4 expression (Figure S1A–C). In the positive control, positive staining was observed exclusively in the cytoplasm of testicular germ cells, primary spermatocytes, secondary spermatocytes, early spermatids, and Leydig cells, as previously described [27,37]. EGR4 expression was not detected in Sertoli cells (Figure S1A–C). No EGR4-immunolabelled cells were observed in the negative control with omission of the primary antibody (Figure S1D–F). These results indicated that the rabbit polyclonal anti-EGR4 primary antibody employed was specific and the biotinylated goat anti-rabbit IgG secondary antibody displayed no discernible cross-reactivity with non-target proteins in mice testis.

**Figure 1:**
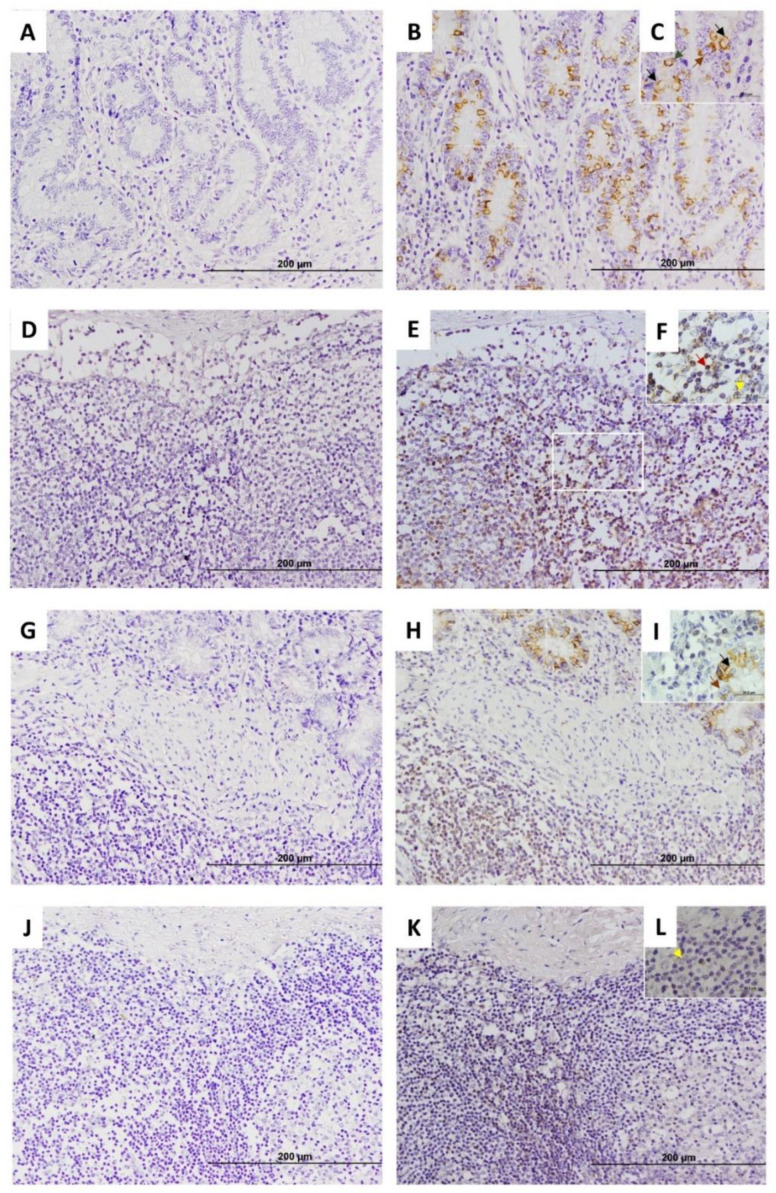
Early growth response factor 4 (EGR4) expression in each tissue section in a Holstein Friesian cow with multifocal lesions using immunohistochemistry. (**A**,**D**,**G**,**J**) represent negative controls carried out with omission of anti-EGR4 primary antibody in distal jejunum (DJE), caudal jejunal lymph node (JELN), ileocecal valve (ICV), and ileocecal lymph node (ICVLN), respectively; (**B**,**E**,**H**,**K**) show positive controls in basal areas of the lamina propria of DJE, cortex of JELN, basal areas of the lamina propria and lymphoid tissue of ICVLN, and cortex area of ICVLN, respectively. Sections were examined at 200× magnification; (**C**,**F**,**I**,**L**) depict magnified areas (1000×) of (**B**,**E**,**H**,**K**) (white squares), respectively. Brown arrows point to type 1 EGR4-positive cells (enterocytes) and dark red arrows point to type 2 cells (lymphocytes). Black arrows point to type 3 EGR4-positive cells (goblet cells) in DJE and ICV. Green arrows point to type 4 EGR4-positive cells (argentophilic or enteroendocrine) in DJE and yellow arrows to type 5 EGR4-positive macrophages in JELN. EGR4 immunostaining was carried out using an anti-EGR4 (LS-B1525, LifeSpan Bioscience, Seattle, WA, USA) antibody and ABC complex.

The IHC procedure was also initially checked in bovine experimental samples of a cow with multifocal lesions. No unspecific staining was observed when the anti-EGR4 primary antibody was omitted (Figure 1A,D,G,J), while EGR4-specific staining was observed in DJE, JELN, ICV, and ICVLN samples when the primary antibody was included (Figure 1B,C,E,F,H,I and K,L, respectively), indicating that the procedure of the anti-EGR4 IHC was working correctly in the four tissue sections under study. The same results were observed in animals with other types of PTB-associated histological lesions.

### 3.3. Morphological Analysis and Distribution Pattern of EGR4-Expressing Cells in Cattle Gut Tissues and Regional Lymph Nodes

**Figure 2:**
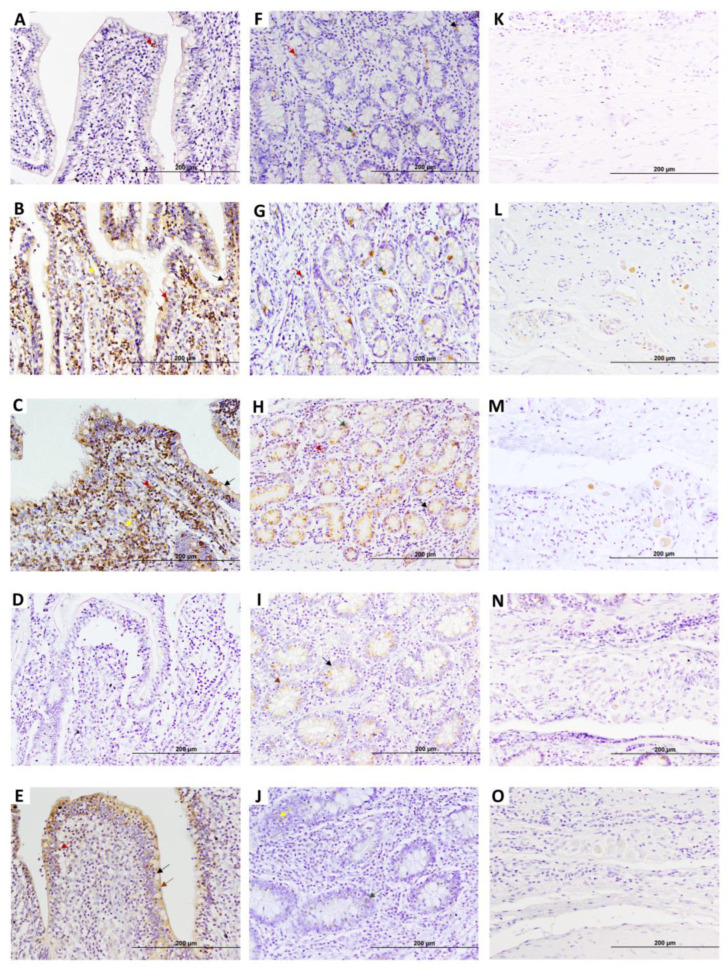
Representative images showing early growth response 4 factor (EGR4) expression in distal jejunum (DJE) of infected cows with different types of PTB-associated histological lesions and control cows without observed lesions. A to E show villi apical areas of DJE, F to J show the basal area of lamina propria where Lieberkhün crypts are more abundant, and K to O show the submucosa area. (**A**,**F**,**K**) control cow with no lesions detected; (**B**,**G**,**L**) cow with PTB-associated focal lesions; (**C**,**H**,**M**) subclinical cow with multifocal lesions; (**D**,**I**,**N**) cow with diffuse intermediate lesions; and (**E**,**J**,**O**) cow with diffuse multibacillary lesions in their intestinal tissues. Original magnification: 200×. Brown arrows point to type 1 EGR4-positive cells (enterocytes), dark red arrows point to type 2 EGR4-positive cells (lymphocytes), and black arrows point to type 3 EGR4-positive cells (goblet cells) in DJE. Green arrows point to type 4 EGR4-positive cells (argentophilic or enteroendocrine cells). Yellow arrows point to type 5 EGR4-positive macrophages in DJE. EGR4-positive staining is mainly detected in both the apical and basal zone of the DJE mucosa, which appears to be more abundant and apically distributed in animals with focal lesions and multifocal lesions without clinical signs. EGR4 immunostaining was carried out using an anti-EGR4 (LS-B1525, LifeSpan Bioscience, Seattle, WA, USA) antibody and ABC complex.

Five EGR4-positive cell types (type 1–5) were observed in the lamina propria of DJE (Figure 1B,C and Figure 2). Type 1 and 2 were cells that were forming part of the villi or surrounding the villi (Figure 2B,C,E). Morphologically, type 1 (Figure 1C) was identified as an epithelial cell (enterocyte) with abundant cytoplasm, a nearly rectangular shape, and medium-sized nuclei (diameter 4.17–9.23 µm); type 2 (Figure 2B,C) corresponded to lymphocytes with small nuclei (diameter 4.06–5.89 µm) and sparce cytoplasm randomly scattered throughout the lamina; type 3 (Figure 1C) was identified as goblet cells with large vacuoles and large, inconspicuous nuclei (diameter 5.52–7.54 µm) that were found in the crypts of Lieberkühn in the most basal part of the lamina propria; type 4 (Figure 1C) corresponded to round medium-sized cells with cytoplasm compatible with enteroendocrine cells or argentaffin cells (diameter 3.68–5.02 µm); and type 5 (Figure 2B,C) were cells with a medium-sized round to oval nuclei with more abundant cytoplasm, similar to macrophages. In DJE (Figure 2), EGR4-positive cells were detected in all animals in a highly variable manner, both in the apical and basal mucosa of DJE (Figure 2A–E and F–J, respectively). In the most apical area of villi, positive cells (enterocytes) were more abundant and formed part of the epithelium (Figure 2B,C,E) and were scattered in the lamina propria. In the basal mucosa, as previously mentioned, EGR4-positive cells were observed to be part of the crypts of Lieberkühn (Figure 2F–J), and in associated lymphoid tissue. In the submucosa, EGR4-positive cells were restricted to Meissner’s submucosal plexus cells (Figure 2K–O). Apparently, observing these images, EGR4-positive cells seemed to be more abundant in animals with focal and multifocal lesions, while the number tended to be lower in animals with intermediate diffuse lesions, slightly increasing in animals with multibacillary diffuse lesions. A small number of positive cells were observed in control animals, showing the same distribution pattern.

**Figure 3:**
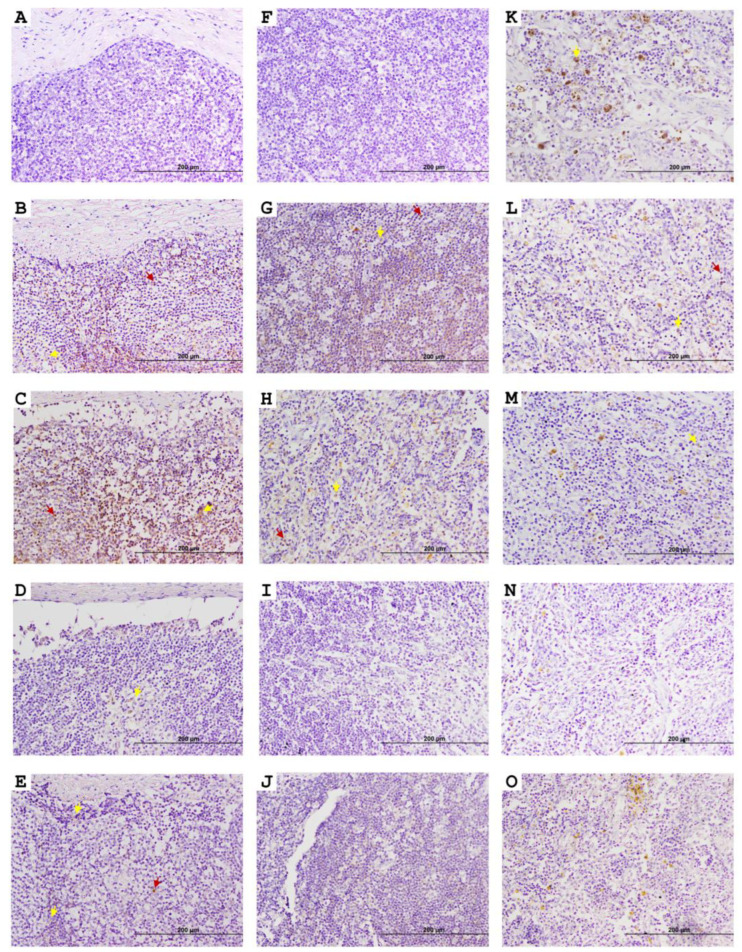
Representative images showing early growth response 4 factor (EGR4) expression in caudal jejunum lymph nodes (JELNs) of infected cows with different PTB-associated histological lesions and control cows without observed lesions. (**A**–**E**), (**F**–**J**), and (**K**–**O**) show cortex where the presence of PTB granulomas was frequent, paracortex, and medullar areas of the JELN, respectively. (**A**,**F**,**K**) control cow with no lesions detected; (**B**,**G**,**L**) cow with PTB-associated focal lesions; (**C**,**H**,**M**) subclinical cow with multifocal lesions; (**D**,**I**,**N**) cow with diffuse intermediate lesions; and (**E**,**J**,**O**) cow with diffuse multibacillary lesions in their intestinal tissues. Original magnification: 200×. EGR4-positive staining was predominantly observed in the cortex of the JELN of animals with focal lesions and multifocal lesions without clinical signs. However, as the disease progresses, the number of EGR4-expressing cells appeared to decrease, eventually becoming nearly undetectable. Dark red arrows point to type 2 cells (lymphocytes) and yellow arrows point to type 5 (macrophages) EGR4-positive cells. EGR4 immunostaining was carried out using an anti-EGR4 (LS-B1525, LifeSpan Bioscience, Seattle, WA, USA) antibody and ABC complex.

In JELNs (Figure 1E,F and Figure 3), two types of EGR4-positive cells were mainly identified. These include the previously mentioned type 2 (Figure 1F) cells, with round nuclei (3.25–5.66 µm) occupying almost all the cytoplasm, compatible with lymphocytes, and type 5 (Figure 1F) cells, with more abundant cytoplasm and oval nuclei (diameter 5.02–8.12 µm), compatible with macrophages. These two cell types were present in all samples (n = thirty nine), with no morphological differences observed between animals of different groups and ages. The EGR4-positive cells were mainly found in the cortex without being part of granulomas, surrounding lymphoid follicles, and sometimes in the proximities of the germinal centre (Figure 3A–E). Some positive cells were also observed in the paracortex area (Figure 3G,H) and occasionally in the medullar area (Figure 3K–O), although they represented a minority compared to those present in the cortex. The number of EGR-4-positive cells appeared to be more abundant towards the afferent lymphatic vessels in both infected and uninfected animals. As in the case of DJE samples, the number of EGR4-positive cells appeared to be higher in animals with focal and multifocal PTB-associated lesions.

**Figure 4:**
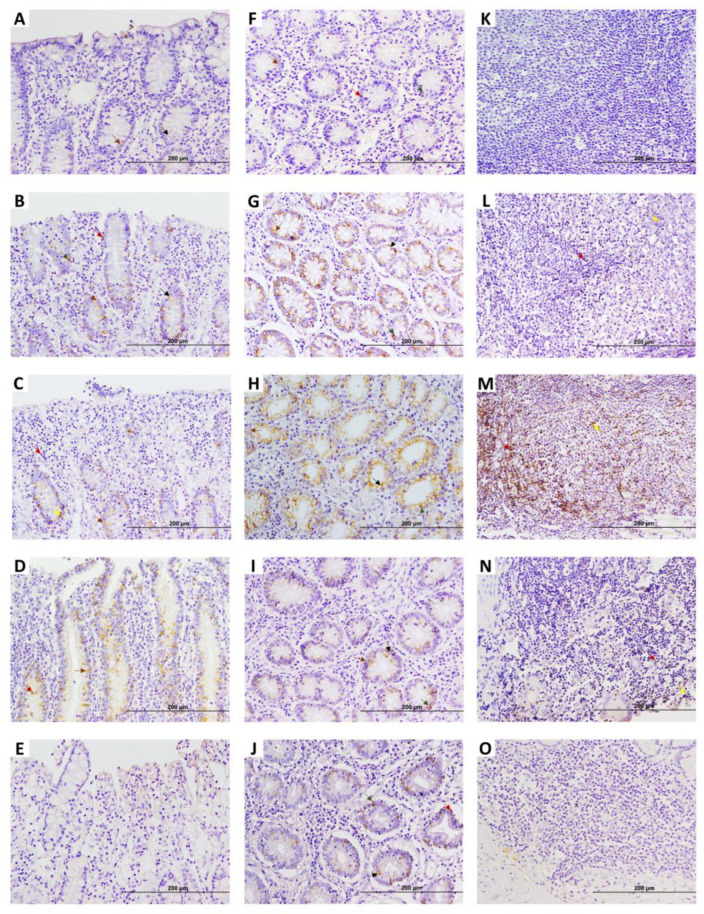
Early growth response 4 (EGR4) factor expression in ileocecal valve (ICV) of infected cows with different PTB-associated histological lesions and control cows without observed lesions. A to E show apical areas of lamina propria of the ICV: (**A**) control cow with no lesions detected; (**B**–**E**) animals with focal lesions, multifocal lesions without clinical signs, diffuse intermediate lesions, and diffuse multibacillary lesions in their intestinal tissues, respectively. (**F**–**J**) display basal area of lamina propria of ICV: (**F**) control cow with no lesions detected; (**G**–**J**) animals with focal lesions, multifocal lesions without clinical signs, diffuse intermediate lesions, and diffuse multibacillary lesions in their intestinal tissues, respectively. (**K**–**O**) show lymphoid tissue areas of ICV: (**K**) control cow with no lesions detected; (**L**–**O**) animals with focal lesions, multifocal lesions without clinical signs, diffuse intermediate lesions, and diffuse multibacillary lesions, respectively. Original magnification: 200×. EGR4-positive staining is mainly detected in the basal region of the ICV, which in the case of animals with focal lesions and multifocal lesions without clinical signs, appears to become more apical and abundant. Yellow arrows point to type 5 (macrophages) EGR4-positive cells Dark red arrows point to type 2 cells (lymphocytes). Black arrows point to type 3 EGR4-positive cells (goblet cells). Green arrows point to type 4 EGR4-positive cells (argentophilic or enteroendocrine cells). Brown arrows point to type 1 EGR4-positive cells (enterocytes).

Regarding the results of the EGR4-IHC analysis in ICV samples (Figure 4), as in the case of DJE, five types of EGR4-positive cells were detected, with a similar tissue distribution. EGR4-positive cells were detected in all animals in a highly variable manner in both the apical and basal mucosa of ICV, although the expression appeared to be present mostly in the basal area (Figure 4A–J). In the apical mucosa, EGR4-positive cells formed part of epithelium (Figure 4D) and were scattered in the lamina propria without being part of granulomas (Figure 4A–E). In the basal mucosa, positive cells were observed to be part of the crypts of Lieberkühn (Figure 4F–J) and confined to lymphoid tissue where expression increased, especially in animals with multifocal lesions (Figure 4K–O). The same observations were made in the control animals, with a markedly lower number of EGR4-positive cells in comparison with animals with focal and multifocal lesions, whilst in animals with intermediate and multibacillary diffuse lesions, the number tended to decrease.

**Figure 5:**
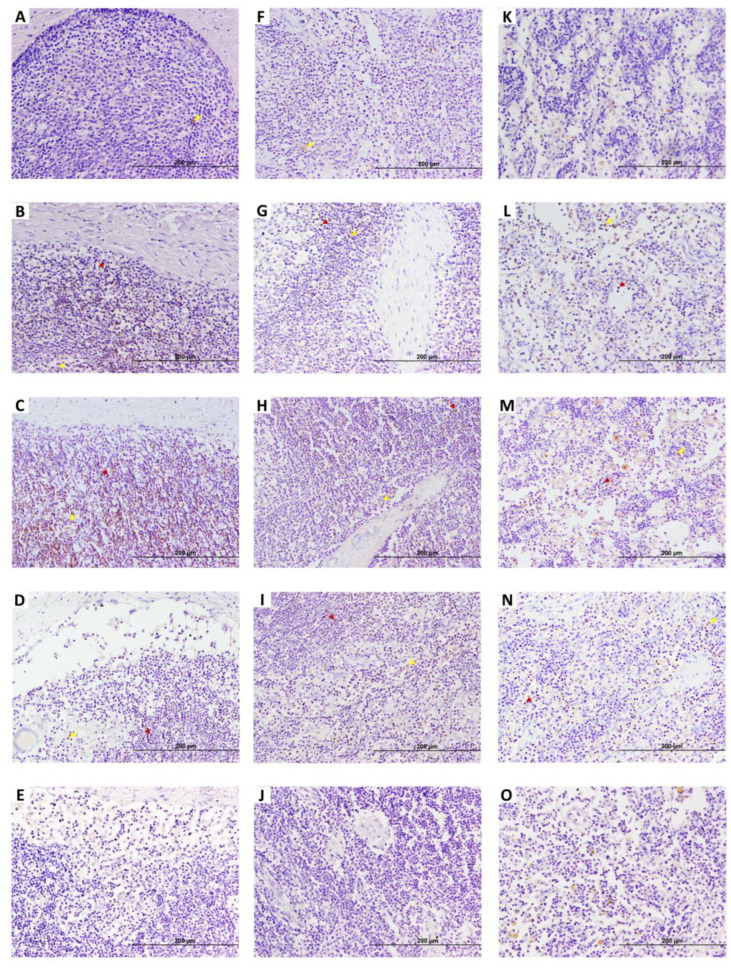
Early growth response 4 (EGR4) factor expression in ileocecal lymph node (ICVLN) samples of infected cows with different histopathological forms of bovine paratuberculosis and control cows without observed lesions. (**A**–**E**), (**F**–**J**), and (**K**–**O**) show cortex (where the presence of PTB-associated granulomas is frequent), paracortex, and medullar areas of the ICVLN, respectively. (**A**,**F**,**K**) control cow with no lesions detected; (**B**,**G**,**L**) cow with PTB-associated focal lesions; (**C**,**H**,**M**) subclinical cow with multifocal lesions; (**D**,**I**,**N**) cow with diffuse intermediate lesions; and (**E**,**J**,**O**) cow with diffuse multibacillary lesions in their intestinal tissues. Original magnification: 200×. EGR4-positive staining is mostly detected in the cortex of the ICVLN of animals with focal lesions and multifocal lesions without clinical signs. Dark red arrows point to type 2 cells (lymphocytes) and yellow arrows point to type 5 (macrophages) EGR4-positive cells. EGR4 immunostaining was carried out using an anti-EGR4 (LS-B1525, LifeSpan Bioscience, Seattle, WA, USA) antibody and ABC complex.

In ICVLNs (Figure 5), similar to JELNs, type 2 (lymphocytes, 3.28–5.42 µm in diameter) and type 5 (macrophages, 5.18–10.44 µm in diameter) (Figure 1L) EGR4-positive cells were identified. These two cell types were also observed in all samples (n = thirty nine) in a variable number, but with a markedly lesser degree of expression compared to the other tissue sections analysed. No morphological differences were identified between the positive cells observed among animals of different groups and ages. As in the case of JELN samples, EGR4-positive cells were observed mainly in the cortex without being part of the granulomas (Figure 5A–E), although some positively labelled cells were observed in the paracortex (Figure 5F–J) and in the medullar area (Figure 5K–O). The number of EGR4-positive cells appeared to be more abundant towards the afferent lymphatic vessels in both infected and uninfected animals. As was observed in the other tissue samples, it appears that the number of EGR4-positive cells seemed to be higher in animals with focal and multifocal lesions, while in the case of animals with intermediate and multibacillary diffuse lesions, the number seemed to be low.

The analysis of the images showed that EGR4 expression was higher in animals with focal and multifocal PTB-associated lesions, with no EGR4-positive cells observed in granulomas.

### 3.4. Quantification of EGR4-Expressing Cells in Animals with Different PTB-Associated Histological Lesions

The number of EGR4-expressing cells per µm^2^ were counted for each animal in ten randomly selected fields (representative of the different tissue areas; for instance, for ICV, this includes the apical and basal laminal propria and lymphoid tissue areas) of each of the four different tissue sections (ICV, DJE, ICVLN, and JELN) for the four or six groups or categories under study. The results, evaluated and quantified individually in each tissue section (ICV, DJE, ICVLN, and JELN) and jointly in all four sections simultaneously for each group under study, are shown in Table 2 and Table 3.

Regarding the total count of the number of EGR4-expressing cells per µm^2^ in the four gut tissue sections (ICV + ICVLN + DJE + JELN) considering six categories of animals, significant higher numbers of EGR4-expressing cells per µm^2^ were found in animals with multifocal lesions without clinical signs in comparison to animals with focal lesions, multifocal lesions with clinical signs, diffuse intermediate lesions, diffuse multibacillary lesions, and control animals without lesions (Table 2). When four categories were considered, significant differences were found between the multifocal group and the control and diffuse groups. In this case, although the number of EGR4-positive cells were higher in animals with multifocal lesions, no significant differences were observed between the multifocal and the focal group. Control animals and animals with diffuse intermediate lesions showed the lowest numbers of EGR4-expressing cells.

Comparing tissue sections individually, higher numbers of EGR4-expressing cells per µm^2^ were observed in DJE and JELNs than in ICV and ICVLNs (Table 3). Considering four categories of animals, DJE animals with multifocal lesions showed significantly higher numbers of positive cells than the control, focal, and diffuse groups, while considering the six categories, animals with multifocal lesions without clinical signs had higher counts than controls, animals with focal lesions, diffuse intermediate lesions, and with multifocal lesions with clinical signs, although no significant differences were observed between both multifocal groups. Animals in the diffuse multibacillary group showed higher numbers than the animals with multifocal lesions without clinical signs, although no significant differences were detected. In JELNs, when four categories were analysed, significant differences were found between the multifocal, diffuse, and control groups, while when six categories were considered, animals with multifocal lesions without clinical signs showed significant differences with the other five groups. In ICV, although EGR4 expression was higher in animals with multifocal lesions, significant differences were only observed between animals with multifocal lesions without clinical signs and animals with diffuse intermediate lesions. In ICVLNs, animals with multifocal lesions did not show significant differences with respect to the rest of the groups, showing lower numbers of EGR4-expressing cells than the control and focal groups. Significant differences between infected and non-infected animals were noted in DJE and JELNs.

With respect to age (see Table 1 and Table 2), the age of animals with multifocal lesions without clinical signs (7.44 ± 2.07 years of age) was significantly higher than that of animals in the remaining groups (Dunn post hoc test, *p* < 0.001 in all cases). No significant differences were observed between the focal and the two diffuse groups, and between the control and the multifocal lesions with clinical signs groups. When we analysed four groups (control, focal, multifocal, and diffuse) significant differences were found between the multifocal, focal, and control group (Dunn post hoc test, *p* < 0.001), but not between the multifocal and diffuse groups.

To investigate the effect of age in the results, a linear model was constructed to predict the number of positive cells per µm^2^ as a function of the histopathological group and age (Table 4). A multivariate model using both variables (age and histopathological group) as predictors, with the effect of the group adjusted by age to avoid bias due to this variable, was constructed. In the multivariate model, both including four or six categories, all the groups had significant and negative coefficients with respect to the multifocal or multifocal without clinical signs reference groups, indicating that they had significantly lower numbers of EGR4-labelled cells per µm^2^ than the reference group. The control group without lesions showed the highest difference with the multifocal reference group (multivariate coefficient −1.41 indicating that the expression is 1.41 units lower than that in the multifocal group without clinical signs). When the six categories were analysed, only the variate “group” had an effect in EGR4 expression, and no significant effect with respect to “age” was observed (*p* = 0.729). A low statistical significance was observed regarding age (multivariate coefficient 0.07 indicating that the expression is 0.07 higher for each unit/extra-year of age) when the four categories were considered in the model.

## 4. Discussion

In this study, the relationship between EGR4 expression, Map infection, and resilience to PTB was investigated. To our knowledge, this is the first analysis of the expression and distribution pattern of EGR4-expressing cells in gut tissues of Holstein Friesian cows with different types of PTB-associated lesions.

In our study, EGR4 expression was upregulated in the intestine (ICV + ICVLN + DJE + JELN) of Map-infected animals (Table 2). Map-infected animals had significantly higher levels of EGR4 expression than uninfected control animals, suggesting that EGR4 expression in the intestine may be a related feature of Map infection. EGR4 expression was mainly enriched in gut tissues of subclinical cattle with PTB-associated multifocal lesions. Animals with multifocal lesions without clinical signs had significantly higher (*p* < 0.001) total counts of EGR4-expressing cells/µm^2^ in gut tissues (DJE + JELN + ILV + ILN) than the other five groups (Table 2), which was independent of age (Table 4). These results indicate that EGR4 expression increases due to Map infection; however, it is not related to lesion severity, as there was no increase in the number of EGR4-expressing cells as the lesion severity increased. In fact, animals with diffuse intermediate lesions showed the lowest number of EGR4-expressing cells in their gut tissues. Within the multifocal histopathological group, differences in the expression levels of EGR4 were observed between subclinical and clinical animals. It seems that animals with multifocal lesions with higher levels of EGR4-expressing cells in their gut tissues were able to control infection and lived longer (7.44 ± 2.07), while animals with multifocal lesions with lower numbers of EGR4-expressing cells were not able to control disease progression, developed clinical signs, and were sacrificed at an earlier age (3.46 ± 1.37). Animals with diffuse lesions and low levels of EGR4-expressing cells were not able to contain the infection effectively; they developed clinical signs and were sacrificed earlier (5.92 ± 1.86). Map-infected animals with focal lesions also live quite long (6.31 ± 2.15), but not because they are tolerant to PTB; they had recent infections or latent forms of Map infection. Control animals were sacrificed at a young age (3.45 ± 2.61) for reasons other than PTB. There seems to be a disconnection between histological lesions and clinical evolution, since animals with histologically similar lesions (multifocal lesions) had different clinical responses (subclinical and clinical). These findings suggest that the number of EGR4-expressing cells have an important effect on the development of clinical disease. The mechanisms that allowed this control need to be further investigated, but we hypothesize that subclinical animals with multifocal lesions are “resilient” to clinical PTB. EGR4 might promote resilience to PTB in multifocal animals without clinical signs through three mechanisms that have been described in the literature for EGR4: (i) limiting NF-kβ-mediated pro-inflammatory response, (ii) controlling tissue damage, acting as a brake on T-cell proliferation and cytokine production, and (iii) favouring tissue repair through interaction with epidermal growth factor receptor (EGFR).

EGR4 could be contributing to the mitigation of the impact of PTB in the host, slowing disease progression and playing a relevant role in disease control by modulating immune responses and promoting tissue repair. It has been found to be significantly upregulated together with other members of the EGR family in the anterior yolk sac of *Mycobacterium marinum*-infected zebrafish embryos, supposedly where some *M. marinum* has been localized, and there is neutrophil production, suggesting that EGRs can be related to neutrophil activation [39]. EGR4 has also been found in human astrocytoma cells infected with Venezuelan equine encephalitis virus (VEEV), where its upregulation was partially dependent on EGR1 at the transcription level [40], and also in cells infected with coronaviruses such as gamma coronavirus infectious bronchitis virus (IBV), alpha coronaviruses, porcine epidemic diarrhea virus (PEDV), and human coronavirus-229E (HCoV-229E), as well as in chicken embryos infected with IBV [41]. This expression was associated with the mediation of inflammation and cell death processes. In the case of coronavirus infection, the upregulation of EGR family genes, in particular EGR1, appears to play a role in regulating viral replication, apoptosis, and the antiviral immune response. Like the other members of the EGR factors, EGR4 exhibits divergent functional regions capable of interacting with a wide range of gene promoter domains. These include genes such as insulin-like growth factor II, EGFR, Transforming Growth Factor (TGF), NF-kB, and the nuclear factor of activated T cells (NFATs) [32,34,42,43,44]. Despite these associations, the specific functions of EGR4 are still not well-understood. However, it has been reported that EGR4 may function as an upstream regulator of several transcription factors involved in cell proliferation and differentiation [27,45,46], thus playing a significant role in processes such as the modulation of the immune response, inflammation, and tissue regeneration [32,33,34].

Regarding the EGR4-expressing cell types and their distribution pattern, our study identified five types of EGR4-positive cells in the lamina propria of DJE and ICV (enterocytes, lymphocytes, macrophages, and goblet and argentaffin cells). The observation of EGR4 expression in epithelial cells and lymphocytes is in agreement with previous studies [29,47,48]. In control animals, EGR4 expression was mainly observed in goblet cells in the crypts of Lieberkühn, which are important structures for cellular processes involved in the maintenance of epithelial tissues (cell growth, renewal, and tissue repair), while the presence of positive cells in the most apical zone during Map infection might suggest a shift towards a more specialized EGR4-role, such as inflammation mediation, activation of immune responses, and the re-establishment of barrier functions, even tissue repair. Therefore, EGR4 could be playing an important role in inflammation, the immune response, and repair process of PTB-associated lesions. During PTB progression, the intestinal tissue suffers from thickening and chronic inflammation, processes which are closely linked to the activation of proinflammatory cytokines as part of the immune response to Map infection. As previously mentioned, the NF-κB protein p50 is an EGR4 interaction partner [32], with NF-κB playing a key role in inflammation and the immune response. This interaction could modulate inflammation in Map-infected animals, protecting the tissue and allowing its repair. In this sense, a novel, shortened splice variant of this transcription factor (EGR4-S) was recently found in breast cancer tissue, which showed an altered cell signalling pathway of the human epidermal growth factor receptor 2 (HER2) pathway, but not in normal breast tissue [49]. Under normal conditions, HER2 helps to regulate cell growth and tissue repair. However, in breast cancer, when there is an overexpression or amplification of the HER2 gene, the receptor produces a signal for excessive cell proliferation that contributes to the development of cancer. In the PTB setting, an increase in epithelial cell proliferation may be positive if it promotes the tissue renewal or repair of Map-infected tissues as part of a protective mechanism or the initiation of tissue remodelling. The HER2 (also referred to as ErbB2) protein has also been observed to be involved in cell migration and the pathogenic invasion of *Mycobacterium leprae* [50].

During tissue repair and fibrogenesis, which involves fibroblast proliferation and matrix synthesis, EGR4 plays a critical role. It has been implicated in fibroblast activity within pathological contexts, particularly in human cancer-associated fibroblasts, where its presence is linked to increased cancer cell proliferation and enhanced extracellular matrix (ECM) remodelling [30]. It is well-established that Map-induced granulomatous processes can trigger tissue fibrosis as part of the repair process. Curiously, sustained EGR4 expression in primary chicken embryo dermal myofibroblasts was shown to disrupt autocrine TGF-β signalling and suppressed the myofibroblastic phenotype, evidenced by the loss of alpha-smooth muscle actin fibres and a marked reduction in ECM production, resulting in an antifibrotic effect [43]. This observation suggests a potential antifibrotic role for EGR4 that would allow it to regulate ECM organisation. ECM regulation was signalled as a key mechanism of tolerance in infectious diseases [15].

Regarding EGR4 expression and the distribution pattern in lymphoid tissues, at least two types of EGR4-positive cells have been identified in the cortex, sometimes near the germinal centre, and occasionally in the medullary regions of JELNs and ICVLNs, each showing similar distribution patterns. Type 1 cells were morphologically identified as lymphocytes, and type 2 cells as macrophages, displaying a markedly lower level of EGR4-expressing cells compared to other tissue sections. In both lymph nodes, EGR4-positive cells were more abundant towards the afferent lymphatic vessels (cortical expression) in both infected and uninfected animals. This common pattern of expression in both lymph nodes could suggest a relevant mechanism in the immune response and inflammation associated with PTB. However, since expression in these tissue sections was low, it seemed to be of less importance or be part of a synchronized response in the intestinal tissue. In control animals, in both JELNs and ICVLNs, EGR4 expression was weak in the lymph node cortex. The presence of EGR4-positive cells mainly detected in the most apical areas during Map infection of animals with focal and multifocal lesions might suggest processes of cell migration to specific areas, where lymphocytes and macrophages could be moving to interact with Map and other cells of the immune system. This migration could be regulating the activation and differentiation of immune cells, since in the peripheral areas of the lymph node, B and T lymphocytes are activated and proliferate as part of the immune response to infection. These processes could be modulating inflammatory mechanisms, since it is well-established that immune modulation and inflammation are closely related [51].

In general, EGR factors play a crucial role in the regulation of immune responses through their influence on lymphocyte precursor differentiation, T and B cell activation, and their involvement in both central and peripheral tolerance processes [52]. Mookerjee-Basu et al. (2020) [33] showed that EGR4 upregulation might result in a regulated NFAT activation, resulting in a critical “brake” on T-cell activation and differentiation. These authors showed that EGR4 may play a negative regulatory role in the expression of IFN_Ɣ_, regulating cytokine production. Moreover, EGR4 acts to reduce the generation of effector T (Teff) cells in general and Th1 cells, in which EGR4 plays a key role in Th polarization, limiting the strength and duration of NF-κβ activation, making T cells poised to respond efficiently to further stimulation. Therefore, it is possible that the expression observed in animals with multifocal lesions can limit the NF-κβ-induced proinflammatory immune response to Map infection controlling the inflammation, resulting in the induction of an anergy/exhaustion stage, and allowing a long-term association with the host, as was hypothesized by [19]. Curiously, most animals with multifocal lesions and a greater number of EGR4-positive cells/µm^2^ were asymptomatic and died or were sacrificed at older ages compared to symptomatic animals from the same multifocal histological group (Table 2 and Table 3).

The current understanding of EGR4 and its role in healthy and Map-infected cattle is limited. The findings of the present study suggest that EGR4 may play a crucial role in the modulation of the molecular mechanisms potentially responsible for PTB resilience. EGR4 may act through some immunoregulatory mechanism that determines the evolution of the disease process. EGR4 could be participating in regulatory mechanisms modulating inflammation and promoting tissue repair in the tissue surrounding the granuloma without modifying the morphology of the granuloma itself. This would explain why, despite having similar histological lesions, some animals control the disease and others do not. However, similar studies including a higher number of animals will be necessary to confirm this hypothesis. Additionally, specific in vitro assays to demonstrate the proposed interactions are required to further elucidate the role of EGR4 in PTB resilience. Once the multiple roles of EGR4 in PTB resilience have been demonstrated, its use as a selective breeding marker could be revolutionary, since the cis eQTL-rs383097118 variant was linked to increased EGR4 expression and the presence of multifocal lesions (SNP effect = 0.222) [19]. This genetic variant can be detected through genomic analysis, and animals with a high EGR4 response can be identified and selectively bred. The implementation of this breeding program would gradually increase the frequency of this resilient allele in the population, which would improve PTB resistance, herd health, and productivity. Therefore, the study of the role of EGR4 in Map-infected cattle may uncover novel strategies for managing the disease, enhancing animal welfare, improving disease resilience, developing new therapeutic treatments, and ultimately, fostering a more sustainable livestock industry.

## 5. Conclusions

This study represents a preliminary evaluation of the role of EGR4 expression in resilience to bovine PTB. The quantitative analysis of EGR4 expression in gut tissues showed that animals with multifocal lesions but no clinical signs had significantly more EGR4-expressing cells in gut tissues and regional lymph nodes. These animals survived longer than the other groups of animals included in the study, revealing a correlation between the upregulation of EGR4 and reduction in disease severity, possibly through the regulation of the immune response. EGR4-expressing cells were identified as enterocytes, lymphocytes, macrophages, argentaffin cells, and goblet cells, mostly in the tissue surrounding the granuloma, but not as part of the granuloma. This distribution pattern of EGR4-expressing cells in the tissue surrounding the granuloma change in Map-infected animals, suggesting a shift towards a more specialized EGR4 role.

## Figures and Tables

**Table 1:** Infection status of control animals and animals with different types of PTB-associated lesions (focal, multifocal, diffuse intermediate, and diffuse multibacillary). The multifocal group was subdivided into two subgroups based on the presence or absence of clinical signs.

ID	ZN	ELISA	PCR-F	PCR-T	Culture-F	Culture-T	Age	CS
Animals with focal lesions (n = 7), age range 2.72–9.48, MEAN ± SD 6.31 ± 2.15								
8	POS (+)	NEG (2.65)	NEG	POS	NEG	POS (<10)	4.71	NO
19	NEG	NEG (9.17)	NEG	POS	NEG	NEG	6.19	NO
31	NEG	NEG (5.51)	NEG	POS	NEG	NEG	5.33	NO
41	NEG	NEG (1.03)	NEG	POS	NEG	POS (<10)	7.15	NO
51	POS (+)	NEG (2.79)	NEG	POS	NEG	NEG	9.48	NO
106	NEG	NEG (2.71)	NEG	POS	NEG	POS	2.72	NO
41F	POS (+)	NEG (6.57)	NEG	POS	NEG	NEG	8.58	NO
Animals with multifocal lesions (n = 12), age range 3.96–10.39, MEAN ± SD 5.80 ± 2.67								
Animals with multifocal lesions without clinical signs (n = 7), age range 3.96–10.39, MEAN ± SD 7.44 ± 2.07								
11	POS (+)	NEG (1.50)	NEG	NEG	NEG	POS (<10)	7.31	NO
48	POS (+)	NEG (4.82)	NEG	NEG	NEG	POS (<10)	9.67	NO
62	POS (+)	NEG (4.58)	NEG	POS	NEG	NEG	10.39	NO
65	POS (+)	NEG (3.46)	NC	POS	NEG	NEG	7.7	NO
76	POS (+)	NEG (4.86)	NEG	POS	NEG	NEG	5.72	NO
95	POS (+)	NEG (5.06)	NEG	POS	NEG	NEG	7.31	NO
140	POS (+)	NEG (14.33)	POS	POS	NEG	NEG	3.96	NO
ID	ZN	ELISA	PCR-F	PCR-T	Culture-F	Culture-T	Age	CS
Animals with multifocal lesions with clinical signs (n = 5), age range 2.48–6.55, MEAN ± SD 3.46 ± 1.37								
46	POS (+)	NEG (2.98)	NEG	NEG	NEG	NEG	2.75	YES
156	POS (+)	NEG (3.37)	NC	NEG	NEG	NEG	6.55	YES
166	POS (+)	NEG (4.49)	POS	POS	NEG	NEG	3.33	YES
213	POS (+)	NEG (3.12)	NEG	POS	NEG	NEG	2.48	YES
97	POS (++)	POS (131.05)	POS	POS	NEG	POS (>50)	2.96	YES
ID	ZN	ELISA	PCR-F	PCR-T	Culture-F	Culture-T	Age	CS
Animals with diffuse lesions, age range 2.92–10.39, MEAN ± SD 5.92 ± 1.86								
Animals with diffuse intermediate lesions, age range 3.87–8.44, MEAN ± SD 5.83 ± 1.47								
25	POS (++)	POS (137.07)	POS	POS	NEG	POS	8.44	ND
141	POS (++)	POS (210.82)	POS	POS	NEG	NEG	3.87	YES
5	POS (++)	POS (205.94)	POS	POS	NEG	POS (>50)	5.22	YES
26	POS (+)	POS (187.69)	POS	POS	NEG	POS (>50)	5.46	NO
59	POS (++)	POS (288.75)	POS	POS	NEG	POS (<10)	7.02	YES
32	POS (+)	POS (241.18)	POS	POS	POS (>50)	POS (>50)	4.47	YES
68	POS (++)	POS (254.72)	POS	POS	POS (>50)	POS	6.01	YES
Animals with diffuse multibacillary lesions, age range 2.92–10.39, MEAN ± SD 6.01 ± 2.17								
92	POS (+++)	POS (174.43)	NC	POS	POS	POS	6.7	YES
101	POS (+++)	POS (242.36)	POS	POS	POS (>50)	POS	5.82	YES
115	POS (+++)	POS (215.95)	POS	POS	NEG	POS (>50)	4.74	YES
88	POS (+++)	POS (286.51)	POS	POS	NEG	POS (>50)	2.92	YES
99	POS (+++)	POS (157.83)	POS	POS	POS (>50)	POS (>50)	4.82	YES
103	POS (+++)	POS (155.82)	POS	POS	NEG	POS (<10)	10.39	YES
116	POS (+++)	POS (169.16)	POS	POS	NEG	POS (10–50)	6.7	YES
Control animals without lesions (n = 6), age range 0.81–8.25, MEAN ± SD 3.45 ± 2.61								
4N	NEG	NEG (5.44)	NEG	NEG	NEG	NEG	3.26	ND
13	NEG	NEG (8.84)	NEG	NEG	NEG	NEG	0.81	NO
12	NEG	NEG (1.72)	NEG	NEG	NEG	NEG	3.58	NO
94	NEG	NEG (1.26)	NEG	NEG	NEG	NEG	2.7	NO
113	NEG	NEG (2.45)	NEG	NEG	NEG	NEG	1.27	NO
7	NEG	NEG (18.37)	NEG	NEG	NEG	NEG	8.25	ND

ZN, Ziehl–Neelsen; The +, ++ and +++, indicate the abundance of the acid-fast bacteria in the sample; ELISA refers to the anti-Map ELISA; Age refers to years; F and T refer to faeces and tissues, respectively; CS, clinical signs; NA, not available; NC, non-conclusive result; the range and the MEAN ± SD of the age is shown for each group and subgroup. For the positive results in the bacteriological cultures, the number of positive colonies is indicated in parenthesis. For the anti-Map ELISA, the result (positive and negative) and the numerical value (percentage of the sample (S)/positive control (P) ratio) obtained in the ELISA are indicated. The cut off value of the ELISA is 55% (S/P ≥55% positive sample). Age refers to the age at which the animals died or were sacrificed due to PTB, accidents in the farm, or other diseases. CS registered were presence of diarrhoea, loss of weight/cachexia, and decreased production of milk. To consider that one animal had clinical signs, it had to present at least two of them.

**Table 2:** Quantification of the total number of EGR4-expressing cells in the four tissue sections (ileocecal valve, distal jejunum, ileocecal lymph nodes, and jejunal lymph nodes) of animals with different types of PTB-associated histological lesions.

	Gut Tissues (DJE + ICV + JELN + ICVLN)		Age
	N	Nº + Cells/µm^2^	
Control	200	0.52 (0.07–0.97) ^a^	2.70 (1.27–3.60) ^a^
Focal	280	0.66 (0.21–1.73) ^b^	6.19 (4.71–8.58) ^b^
Multifocal-WithoutCS	270	1.17 (0.41–2.70) ^c^	7.31 (5.72–9.67) ^c^
Multifocal-WithCS	190	0.61 (0.12–1.57) ^a,b,d^	2.96 (2.75–3.33) ^a^
Diffuse intermediate	260	0.40 (0.05–1.04) ^a,d^	5.46 (4.47–7.02) ^b^
Diffuse multibacillary	280	0.79 (0.05–2.39) ^b,e^	5.82 (4.74–6.70) ^b^
Statistical analysis	*Kruskal–Wallis (p < 0.001)*		*Kruskal–Wallis (p < 0.001)*
Post hoc analysis	Dunn’s test		Dunn’s test
Control	200	0.52 (0.07–0.97) ^a^	2.70 (1.27–3.60) ^a^
Focal	280	0.66 (0.21–1.73) ^b,c^	6.19 (4.71–8.58) ^b^
Multifocal	460	0.95 (0.25–2.24) ^b^	5.72 (2.96–7.70) ^c^
Diffuse	540	0.54 (0.05–1.66) ^a,c^	5.64 (4.74–6.70) ^b,c^
Statistical analysis	*Kruskal–Wallis (p < 0.001)*		*Kruskal–Wallis (p < 0.001)*
Post hoc analysis		Dunn’s test	Dunn’s test
Control	200	0.72 ± 0.85	3.45 ± 2.61
Infected animals	1280	1.49 ± 2.09 ***	5.96 ± 2.25 ***
Statistical analysis	*Welch’s test (p < 0.001)*		*Student-s test (p < 0.001)*

GR4, early growth response factor 4. Multifocal-WithoutCS and Multifocal-WithCS refer to animals with multifocal lesions without and with clinical signs, respectively. Controls refer to control animals without lesions. Infected animals refer to animals with PTB-associated lesions (any type). N, number of fields/images examined per histopathological group. Nº + cells/µm^2^ refers to the number of positive cells expressing EGR4, which are expressed as mean or median values of the number of positive cells per µm^2^ in a field and histopathological group depending on the statistical test used to analyse the data (means ± standard deviation for parametric tests and median (interquartile range P25-P75) for non-parametric tests). Asterisks *** indicate *p*-value < 0.001. The superscript letters indicate the result of the complete post hoc analysis (i.e., all possible pair comparisons). Groups with the same superscript letter are not significantly different from each other. Groups with different letters do show statistically significant differences.

**Table 3:** Quantification of the number of EGR4-expressing cells in each of the four tissue sections analysed (ileocecal valve, distal jejunum, ileocecal lymph nodes, and jejunal lymph nodes) of animals with different types of PTB-associated histological lesions.

		Distal Jejunum	Jejunal LN	Ileocecal Valve	Ileocecal LN
	N	Nº + Cells/µm^2^	Nº + Cells/µm^2^	Nº + Cells/µm^2^	Nº + Cells/µm^2^
Control	40–60	0.21 (0.05–0.64) ^a^	0.42 (0.02–1.07) ^a^	0.65 (0.37–1.32) ^a,b^	0.61 (0.29–0.91) ^a^
Focal	70	0.90 (0.34–2.86) ^b^	0.52 (0.08–1.48) ^a^	0.70 (0.23–1.65) ^a,b^	0.62 (0.14–1.56) ^a,b^
Multifocal-WithoutCS	60–70	2.23 (1.03–4.60) ^c^	2.29 (1.11–4.53) ^b^	0.85 (0.33–1.86) ^b,c^	0.32 (0.12–0.71) ^a,b,c^
Multifocal-WithCS	40–50	1.76 (0.48–3.42) ^b,c^	0.17 (0.03–0.60) ^a^	1.00 (0.37–1.61) ^a,c^	0.33 (0.02–0.96) ^a,b,c^
D. intermediate	60–70	0.70 (0.12–1.84) ^b,c^	0.38 (0.05–0.93) ^a^	0.53 (0.17–0.90) ^a^	0.16 (0.00–0.50) ^c^
D. multibacillary	70	2.65 (0.50–4.28) ^b,c,d^	0.32 (0.01–2.39) ^a^	0.83 (0.38–1.49) ^a,b^	0.26 (0.00–1.36) ^a,b,c^
Statistical analysis		*Kruskal–Wallis (p < 0.001)*	*Kruskal–Wallis (p < 0.001)*	*Kruskal–Wallis (p = 0.027)*	*Kruskal–Wallis (p < 0.001)*
Post hoc test		Dunn’s test	Dunn’s test	Dunn’s test	Dunn’s test
Control	50–60	0.21 (0.05–0.64) ^a^	0.42 (0.02–1.07) ^a^	0.65 (0.37–1.32) ^a^	0.61 (0.29–0.91) ^a^
Focal	70	0.90 (0.34–2.86) ^b^	0.52 (0.08–1.48) ^a,b^	0.70 (0.23–1.65) ^a^	0.62 (0.14–1.56) ^a^
Multifocal	120	1.91 (0.88–4.22) ^c^	1.10 (0.17–2.71) ^b^	0.92 (0.35–1.79) ^a^	0.32 (0.07–0.82) ^a,b^
Diffuse	130–140	1.52 (0.18–3.46) ^b^	0.36 (0.02–1.60) ^a^	0.70 (0.25–1.12) ^a^	0.22 (0.00–0.89) ^b^
Statistical analysis		*Kruskal–Wallis (p < 0.001)*	*Kruskal–Wallis (p < 0.001)*	*Kruskal–Wallis (p = 0.069)*	*Kruskal–Wallis (p < 0.001)*
Post hoc test		Dunn’s test	Dunn’s test	Dunn’s test	Dunn’s test
Control	50–60	0.60 ± 0.89	0.68 ± 0.86	0.93 ± 0.93	0.70 ± 0.63
Infected animals	320–330	2.22 ± 2.19 ***	1.68 ± 2.65 ***	1.1 ± 1.33	0.90 ± 1.67
**Statistical Analysis**		Welch’s test (*p* < 0.001)	Student’s test (*p* < 0.001)	Welch’s test (*p* = 0.242)	Welch’s test (*p* = 0.145

D. refers to diffuse lesions; LN, lymph nodes; EGR4, early growth response factor 4. Multifocal-WithoutCS and Multifocal-WithCS refer to animals with multifocal lesions without and with clinical signs, respectively; D., PTB-associated diffuse lesions. Controls refer to control animals without lesions. Infected animals refer to animals with PTB-associated lesions (any type). N, range of the number of fields/images examined per histopathological group. Nº + cells/µm^2^ refers to the number of positive cells expressing EGR4, which are expressed as mean or median values of the number of immunolabelled cells per µm^2^ in a field and histopathological group depending on the statistical test used to analyse the data (means ± standard deviation for parametric tests and median (interquartile range P25–P75) for non-parametric tests). Asterisks indicate whether the differences between the control group and the infected animals group are significant or not *** *p*-value < 0.001). The superscript letters indicate the result of the complete post hoc analysis (i.e., all possible pairs of comparisons). Groups with the same letter superscript are not significantly different from each other. Groups with different letters show statistically significant differences. Results obtained for animals with multifocal lesions are shown in bold font.

**Table 4:** A multivariate linear model was constructed to predict the number of positive cells per µm^2^ as a function of the histopathological group and age.

Variate	Group	Coefficient (CI 95%, *p*-Value)
GROUP	MULTIFOCAL-WCS	-
	Diffuse intermediate	−1.23 (−1.57 to −0.90, *p* < 0.001)
	WITHOUT LESIONS	−1.41 (−1.81 to −1.01, *p* < 0.001)
	Focal	−0.88 (−1.21 to −0.55, *p* < 0.001)
	Diffuse multibacilary	−0.37 (−0.70 to −0.04, *p* = 0.026)
	MULTIFOCAL-WITHCS	−0.94 (−1.35 to −0.54, *p* < 0.001)
AGE		0.01 (−0.04 to 0.06, *p* = 0.729)
GROUP	Multifocal	-
	Focal	−0.52 (−0.81 to −0.23, *p* < 0.001)
	WITHOUT LESIONS	−0.89 (−1.23 to −0.55, *p* < 0.001)
	Diffuse	−0.41 (−0.65 to −0.16, *p* = 0.001)
AGE		0.07 (0.02 to 0.11, *p* = 0.003)

MEAN ± SD refers to the mean and standard deviation of the quantitative variable (number of EGR4-labelled cells per µm^2^) for GROUP, and the mean value and standard deviation of the age of all the animals included in the study for the variate AGE. MULTIFOCAL-WCS and MULTIFOCAL-WITHCS refer to animals with multifocal lesions without and with clinical signs, respectively. WITHOUT LESIONS refers to the control animals without PTB-associated histological lesions detected. The control without lesions has a negative multivariate coefficient of −1.41 with respect to the reference group (MULTIFOCAL-WCS), indicating that the expression is 1.41 units lower than that in the multifocal group without lesions.

## Data Availability

The original data presented in the study are openly available in ZENODO at https://zenodo.org/records/15113175 (accessed on 17 February 2025).

## Supplementary Materials

The following supporting information can be downloaded at: https://www.mdpi.com/article/10.3390/ani15071012/s1, Figure S1. Immunolocalization of early growth response factor 4 (EGR4) in seminiferous tubes of murine testis. Murine testis is used as a positive control tissue for EGR4 expression. A to C represent positive controls performed without omission of any reagent: (A) EGR4 expression in testicular germ cells, where low or almost no EGR4 expression is observed in primary spermatocytes, and higher levels are detected in secondary spermatocytes and early spermatids, with no EGR4 expression detected in Sertoli cells; (B) closer view of seminiferous tubes; and (C) positive-labelled Leydig cells. D to F show negative controls carried out with omission of the primary antibody: (D) negative labelling in all cells belonging to seminiferous tubes; (E) closer view of seminiferous tubes; and (F) negative labelling of Leydig cells. A and D are at 100×, B and E are at 200×, and C and F are at 400× magnification. In the EGR4-IHC assay, a rabbit polyclonal antibody anti-(C-terminus) EGR4 (LS-B1525, LifeSpan Bioscience, Seattle, WA, USA) was used as primary antibody, ABC complex as conjugate, and 3,3- Diaminobenzidine (DAB) as chromogen (brown color).
