# Supplementary figures and images for: Early Growth Response Factor 4 (EGR4) Expression in Gut Tissues and Regional Lymph Nodes of Cattle with Different Types of Paratuberculosis-Associated Lesions: Potential Role of EGR4 in Resilience to Paratuberculosis
Source: Animals (Basel). 2025 Mar 31;15(7):1012. doi: 10.3390/ani15071012 (PMC11988129; doi:10.3390/ani15071012)

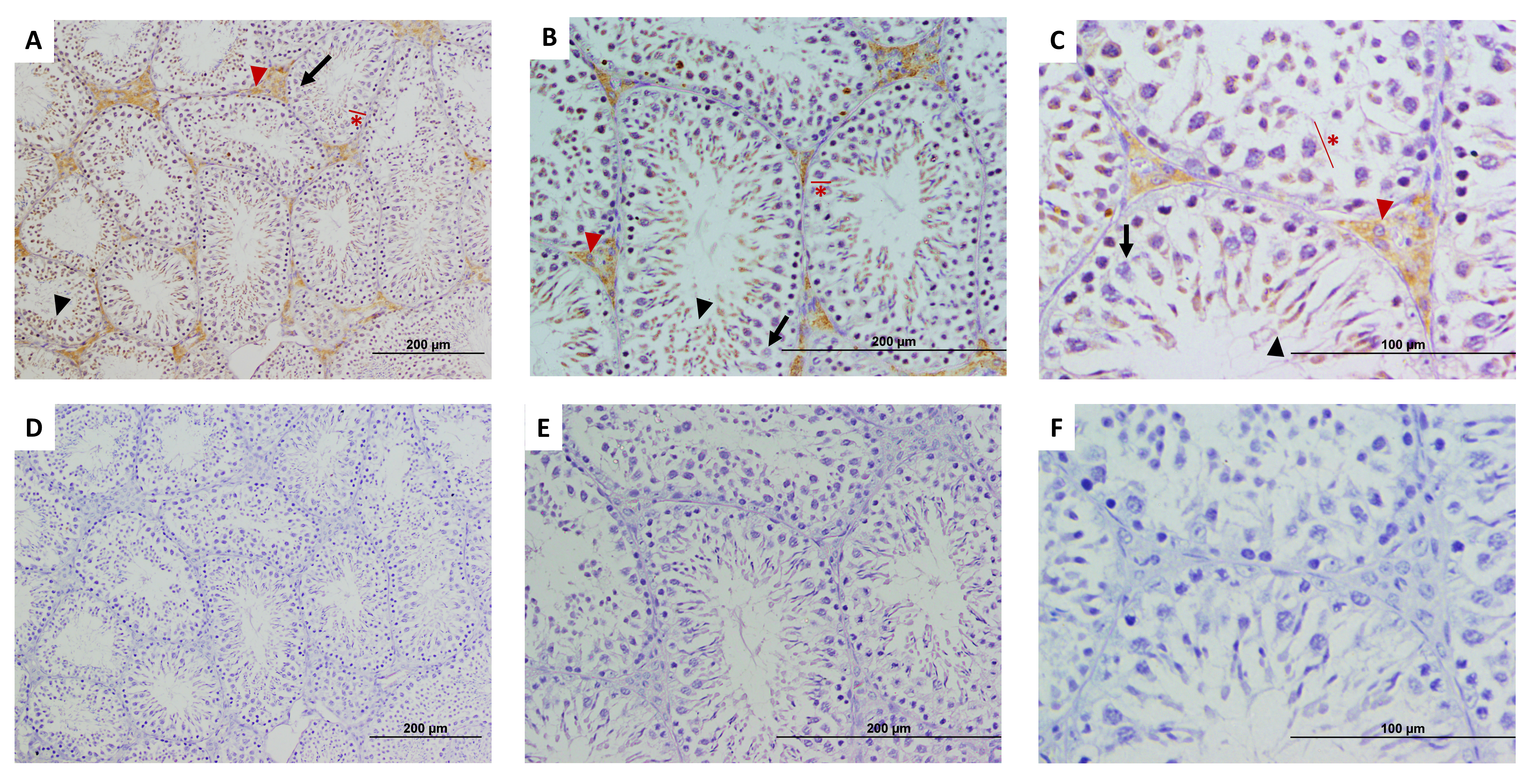

Supplement: Supplementary file 1 [file animals-15-01012-s001.zip › Figure S1.tif]
